# Potential arteriviral spillover: An emerging threat to public health?

**DOI:** 10.3389/fmicb.2023.1156327

**Published:** 2023-03-02

**Authors:** Rui Li, Hongfang Ma, Songlin Qiao, Gaiping Zhang

**Affiliations:** ^1^Key Laboratory of Animal Immunology of the Ministry of Agriculture, Henan Provincial Key Laboratory of Animal Immunology, Henan Academy of Agricultural Sciences, Zhengzhou, China; ^2^School of Physical Education and Health Administration, Henan Finance University, Zhengzhou, China

**Keywords:** zoonotic spillover, cross-species transmission, arterivirus, SHFV, CD163

## Introduction

Zoonotic spillover is one of the most serious threats to public health. Outbreaks of zoonotic viruses, such as avian influenza virus (Miranda et al., 2022) and monkeypox virus (Bragazzi et al., 2022), as well as the ongoing epidemic severe acute respiratory syndrome coronavirus 2 (SARS-CoV-2) (da Silva Torres et al., 2022) highlight their hazards to the global public health and economy.

Arteriviruses are enveloped positive-strand RNA viruses in the *Nidovirale* order along with coronaviruses (Snijder et al., 2013). These viruses have been previously shown to infect equids, mice, swine, and non-human primates (Brinton and Snijder, 2008). Of note, simian hemorrhagic fever virus (SHFV), an arterivirus endangering non-human primates, has been recently reported to be poised for spillover to human *via* an intracellular receptor CD163, which raises an alert on a potential cross-species transmission (Warren et al., 2022). Under One Health, we assessed the potential cross-species transmission of arteriviruses and further evaluated the interspecies receptor usage by arteriviruses to express our concern over the emerging risk of arteriviruses to breach the interspecies boundaries among animals and human.

## Cross-species transmission of arteriviruses

**Figure 1:**
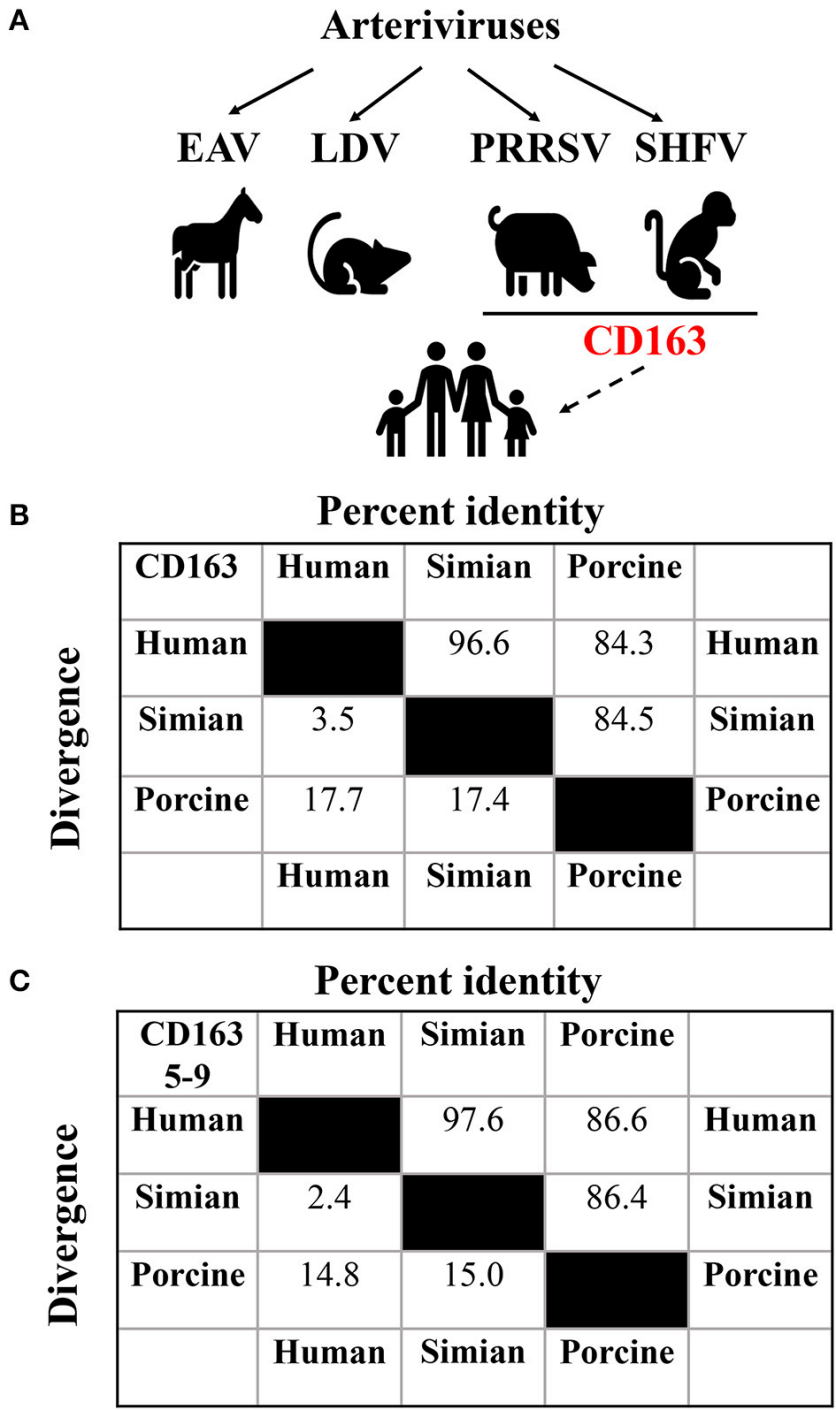
Assessment of potential arteriviral spillover to human. **(A)** Potential cross-species transmission of arteriviruses. The solid lines indicate documented transmission, while the dashed one indicates potential transmission. The putative generic receptor CD163 for SHFV and PRRSV spillover to human is highlighted in red. **(B)** Analysis of the sequence identity among human (UniProt entry Q86VB7), simian (UniProt entry Q2VLG4), and porcine (UniProt entry Q2VL90) CD163 using CLUSTAL W method of DNASTAR Lasergene software (Madison, USA). **(C)** Analysis of the sequence identity among human, simian, and porcine CD163 SRCR5-9 using CLUSTAL W method of DNASTAR Lasergene software as described in **(B)**.

As shown in Figure 1A, arteriviruses include equine arteritis virus (EAV), lactate dehydrogenase-elevating virus (LDV), porcine reproductive and respiratory syndrome virus (PRRSV), and SHFV (Snijder et al., 2013). These viruses establish natural persistent infections in equids (EAV), mice (LDV), swine (PRRSV), and non-human primates (SHFV), and propagate primarily in their respective host macrophages (Brinton and Snijder, 2008). However, certain arteriviruses replicate efficiently in a wide variety of cells without species specificity (Table 1).

**Table 1:** Overview of cell tropisms and host ranges for arteriviruses.

**Arteriviruses**	**Cellular receptor**	**Cell tropism**	**Host**
EAV	CXCL16	Horse macrophages Horse/rabbit/hamster kidney cells BHK-21 RK-13 Vero	Equids
LDV	Not determined	Murine macrophages	Mice
PRRSV	CD163	Porcine alveolar macrophages MA-104 MARC-145	Swine
SHFV	CD163	Simian macrophages MA-104 MARC-145 COS-7 SU-DHL-1 (human)	Non-human primates

For example, EAV is able to proliferate in primary horse, rabbit, and hamster kidney cells as well as various cell lines, such as baby hamster kidney (BHK)-21, rabbit kidney (RK)-13, and African green monkey kidney Vero cells (Payne, 2017). PRRSV is capable of infecting African green monkey kidney epithelial MA-104 and its derivative MARC-145 cells (Kim et al., 1993). In addition to simian macrophages, SHFV has been shown to multiply in MA-104 and MARC-145 cells, whereas it is recently demonstrated to enter into and replicate in human histiocytic SU-DHL-1 cells as described above (Warren et al., 2022). Therefore, arteriviruses possess an inherent potential for dissemination to other cell species.

## Interspecies receptor usage by arteriviruses

As a host cellular receptor usually determines viral cell tropism and even host range, we further evaluated the interspecies receptor usage by arteriviruses based on analyzing the amino acid sequence identities of arteriviral receptors amongst different species.

CD163 is a class I scavenger receptor (SR) containing nine SR cysteine-rich (SRCR) domains (SRCR1-9), and functions in multiple physiological and pathological processes (Van Gorp et al., 2010). Simian CD163 is a crucial cell factor for SHFV entry into MA-104 and MARC-145 cells (Caì et al., 2015), and human CD163 is exploited by SHFV as an intracellular receptor to infect SU-DHL-1 cells (Warren et al., 2022). Therefore, we firstly determined the amino acid sequence identity between human and simian CD163 for SHFV. In Figure 1B, human and simian CD163 share almost identical amino acid sequences (96.6% identity). Since CD163 SRCR5-9 is supposed to be the putative arteriviral interaction domain (Warren et al., 2022), we further conducted a comparative analysis of the amino acid sequences between human and simian CD163 SRCR5-9, and it shows a higher sequence identity (97.6% identity, Figure 1C). These high sequence identities may partly explain why SHFV can utilize human CD163 for transmission to human cells. In the meantime, these analyses remind us of another potential arteriviral spillover by PRRSV, which utilizes porcine CD163 as an indispensable receptor (Whitworth et al., 2016). As mentioned above, PRRSV infects simian CD163-expressed MA-104 and MARC-145 cells as SHFV does (Kim et al., 1993). More importantly, expression of CD163 from different species (including porcine, simian, and human CD163) renders permissiveness to PRRSV infection in numerous refractory cells (Calvert et al., 2007). Interestingly, porcine CD163 only shows 84.5% and 84.3% in sequence identity compared to simian and human versions, respectively (Figure 1B), whereas porcine CD163 SRCR5-9 shows 86.4 and 86.6% in sequence identity compared to simian and human versions, respectively (Figure 1C). These analyses suggest that CD163 can function as a generic receptor for PRRSV to cross cell species despite sequence divergence within a certain range.

Equine CXCL16 has been identified as an entry receptor for EAV in equine cells. Additionally, non-permissive human embryonic kidney (HEK)-293T cells become susceptible to EAV infection after stably expressing equine CXCL16 (Sarkar et al., 2016). However, CXCL16 from different species share a relatively low sequence identity (data not shown). As a consequence, whether CXCL16 is a determinant for EAV broad cell tropism remains to be demonstrated, and there may exist other genuine generic receptors for EAV.

## Discussion

Indeed, the cell tropism of arteriviruses may not be applicable to their *in vivo* infections. Fortunately, infection by arteriviruses hasn't yet documented in human until now. However, three bat-originated coronaviruses, severe acute respiratory syndrome coronavirus, Middle East respiratory syndrome coronavirus, and SARS-CoV-2 have broken the original and intermediate host barriers to infect human upon recognizing human versions of receptors (Cui et al., 2019; Li et al., 2020; Frutos et al., 2021). It is acknowledged that coronavirus evolution *via* genetic mutation contributes to viral adaption to human receptors and cross-species transmission (Cui et al., 2019; Zhai et al., 2020). Considering this fact, we cannot preclude the possibility that SHFV and PRRSV may also evolve to gain a capability of infecting human *via* efficient usage of human CD163. As a consequence, comprehensive investigation on the interaction between SHFV/PRRSV and CD163 from different species is imperative to evaluate their potential spillover to human from the receptor perspective. Structural studies on SHFV/PRRSV proteins in complex with human, simian, or porcine CD163 will be beneficial for understanding receptor hotspots for viral binding and interspecies receptor usage.

In addition to receptors, host cellular restriction factors and immune responses, especially innate immune responses as the first line of defense, have been identified to determine viral cross-species transmission. Therefore, it is required to elucidate whether there exist certain host cellular restriction factors or different host innate immune response levels to prevent arteriviral replication in human.

SHFV causes a fatal hemorrhagic fever in macaque colonies worldwide and primate facilities in several countries. PRRSV is a pathogen of veterinary importance and leads to significant economic losses in the global swine industry. They actually pose an emerging threat to public health for close contact exists among human and arteriviral hosts, exemplified by non-human primate facility workers and infected non-human primates, or pig farmers and sick pigs. As the analyses described above suggest a potential interspecies transmission of arteriviruses, we propose a systemic surveillance in arteriviral hosts and people with close contact to prevent potential outbreaks through serological and virological tests along with other One Health approaches.

## Author contributions

RL and GZ conceived the study. RL and HM conducted the analyses. RL wrote the manuscript. RL, HM, SQ, and GZ revised the manuscript. All authors read and approved the manuscript.
